# LONGITUDINAL RELATIONS OF INTERNET USE FREQUENCY WITH COGNITIVE AND SOCIAL FUNCTIONING AMONG OLDER ADULTS

**DOI:** 10.1093/geroni/igae098.2561

**Published:** 2024-12-31

**Authors:** Wei Xing Toh, Chin Wei Ho, Shuna Shi Ann Khoo

**Affiliations:** Nanyang Technological University, Singapore, Singapore; Nanyang Technological University, Singapore, Singapore; National University of Singapore, Singapore, Singapore

## Abstract

Given the increasing reliance on the Internet in our daily lives, understanding the role of Internet use in cognitive and social functioning among older adults is crucial for promoting successful aging. However, the directionality of these relations remains inconclusive owing to methodological limitations in past studies (e.g., Mariano et al., 2021; Szabo et al., 2019), such as (a) reliance on cross-sectional and limited (two-wave) longitudinal data and (b) failure to disaggregate stable, between-person differences from temporal, within-person dynamics. Drawing from four waves of the English Longitudinal Study of Aging (N = 12,264; Mean age = 66.01 years), we used random-intercept cross-lagged panel modelling to investigate the directionality of Internet use frequency with cognitive functioning (i.e., verbal fluency and episodic memory) and social functioning (i.e., perceived social support, social-interaction frequency, and perceived social closeness). At the between-person level, older adults with higher Internet use frequency reported greater cognitive and social functioning (rs >.11, ps <.001). At the within-person level, greater-than-average cognitive functioning unidirectionally predicted subsequently higher Internet use frequency (βs >.06, ps <.035), thereby indicating that more cognitively intact older adults may face fewer difficulties with Internet use. Conversely, Internet use frequency was bidirectionally associated with social functioning (βs >.11, ps <.001). Specifically, engagement with the Internet supports social networks, and improved social functioning likely enhances the perceived usefulness and usage behavior of the Internet. These findings underscore the importance of considering cognitive and social factors when promoting Internet use among older adults.

